# Congenital emphysematous lung disease associated with a novel Filamin A mutation. Case report and literature review

**DOI:** 10.1186/s12887-019-1460-4

**Published:** 2019-03-29

**Authors:** Gloria Pelizzo, Mirella Collura, Aurora Puglisi, Maria Pia Pappalardo, Emanuele Agolini, Antonio Novelli, Maria Piccione, Caterina Cacace, Rossana Bussani, Giovanni Corsello, Valeria Calcaterra

**Affiliations:** 1 Pediatric Surgery Department, Children’s Hospital “G. di Cristina”, ARNAS Civico-Di Cristina-Benfratelli, Via dei Benedettini, 1, 90134, Palermo, Italy; 2 Cystic Fibrosis and Respiratory Pediatric Center, Children’s Hospital G. Di Cristina, ARNAS Civico-Di Cristina-Benfratelli, Palermo, Italy; 3 Pediatric Anesthesiology and Intensive Care Unit, Children’s Hospital G. Di Cristina, ARNAS Civico-Di Cristina-Benfratelli, Palermo, Italy; 4 Pediatric Radiology Unit, Children’s Hospital G. Di Cristina, ARNAS Civico-Di Cristina-Benfratelli, Palermo, Italy; 5 Laboratory of Medical Genetics, Bambino Gesù Children’s Hospital, Rome, Italy; 6 Department of Sciences for Health Promotion and Mother and Child Care “Giuseppe D’Alessandro”, University of Palermo, Palermo, Italy; 7 Neonatal Intensive Care Unit, Hospital “Barone Romeo” Patti, ASP Messina, Messina, Italy; 8 Institute of Pathological Anatomy, Trieste University Hospital, Trieste, Italy; 9 Pediatrics and Neonatal Intensive Therapy Unit, Mother and Child Department, University of Palermo, Palermo, Italy; 10 Pediatrics and Adolescentology Unit, Department of Internal Medicine University of Pavia and Fondazione IRCCS Policlinico San Matteo, Pavia, Italy

**Keywords:** Filamin a, Congenital enphysema, Lung disease, Children, Periventricular nodular heterotopia

## Abstract

**Background:**

Progressive lung involvement in Filamin A (FLNA)-related cerebral periventricular nodular heterotopia (PVNH) has been reported in a limited number of cases.

**Case presentation:**

We report a new pathogenic *FLNA* gene variant (c.7391_7403del; p.Val2464Alafs*5) in a male infant who developed progressive lung disease with emphysematous lesions and interstitial involvement. Following lobar resection, chronic respiratory failure ensued necessitating continuous mechanical ventilation and tracheostomy. Cerebral periventricular nodular heterotopia was also present.

**Conclusions:**

We report a novel variant of the *FLNA* gene, associated with a severe lung disorder and PNVH. The lung disorder led to respiratory failure during infancy and these pulmonary complications may be the first sign of this disorder. Early recognition with thoracic imaging is important to guide genetic testing, neuroimaging and to define optimal timing of potential therapies, such as lung transplant in progressive lung disease.

## Background

Filamins are large actin-binding proteins that stabilize delicate three-dimensional actin webs and link these to cellular membranes. They integrate cellular architectural and signalling functions and are essential for fetal development and cell locomotion [1].

Filamin A (FLNA) is the first actin filament cross-linking protein identified in non-muscle cells. Mutations in the X-linked gene encoding filamin A (at chromosomal locus Xq28) have been reported to cause a wide range of human diseases, such as cerebral periventricular nodular heterotopia (PVNH), cardiac valvular disease and skeletal anomalies to a variable degree [2–10]. Airway anomalies such as tracheal stenosis or tracheobronchomalacia have also been documented and recently lung involvement has been reported [2, 11–22].

*FLNA*-related PVNH is a malformation of cortical development characterized by bilateral near-contiguous ectopic neuronal nodules found along the lateral ventricles [6, 7]. It may be isolated or associated with other brain malformations, including hippocampal malformation and cerebellar hypoplasia, bilateral fronto-perisylvian or temporo-parietooccipital polymicrogyria, hydrocephalus and microcephaly. In a smaller group of patients, PVNH was found to be associated with non-neurologial defects including Ehlers–Danlos syndrome, frontonasal dysplasia, limb abnormalities, ambiguous genitalia and fragile-X syndrome. Finally, several distinct subgroups of patients have been identified with an unusual PVNH presentation, including a micronodular appearance, unilateral distribution and laminar or ribbon-like shapes [23]. Progressive lung involvement in *FNLA*-related PVNH has been reported in a limited number of cases [2, 11–22] and emphysematous lesions in the pulmonary parenchyma are the characteristic findings of this mutation.

We report the case of a male infant with a novel pathogenic variant of the *FLNA* gene mutation, who developed significant lung disease and in whom a periventricular nodular heterotopia was also diagnosed.

## Case presentation

A 32 day old male infant was referred to our department, from another hospital, with acute respiratory distress syndrome and suspected congenital pulmonary malformation. The baby (fourth child of nonconsanguineous caucasian parents) was born by vaginal delivery at 37 weeks’ gestation, with a weight of 3140 g. The first month of life was unremarkable. The family had no history of genetic or metabolic diseases or congenital disorders.

At admission, the physical examination confirmed respiratory distress, general hypotonia due to respiratory failure and fatigue, bilateral inguinal hernia and deformities of the lower limbs (*pes tortus congenitalis* and hip dysplasia).

**Fig. 1:**
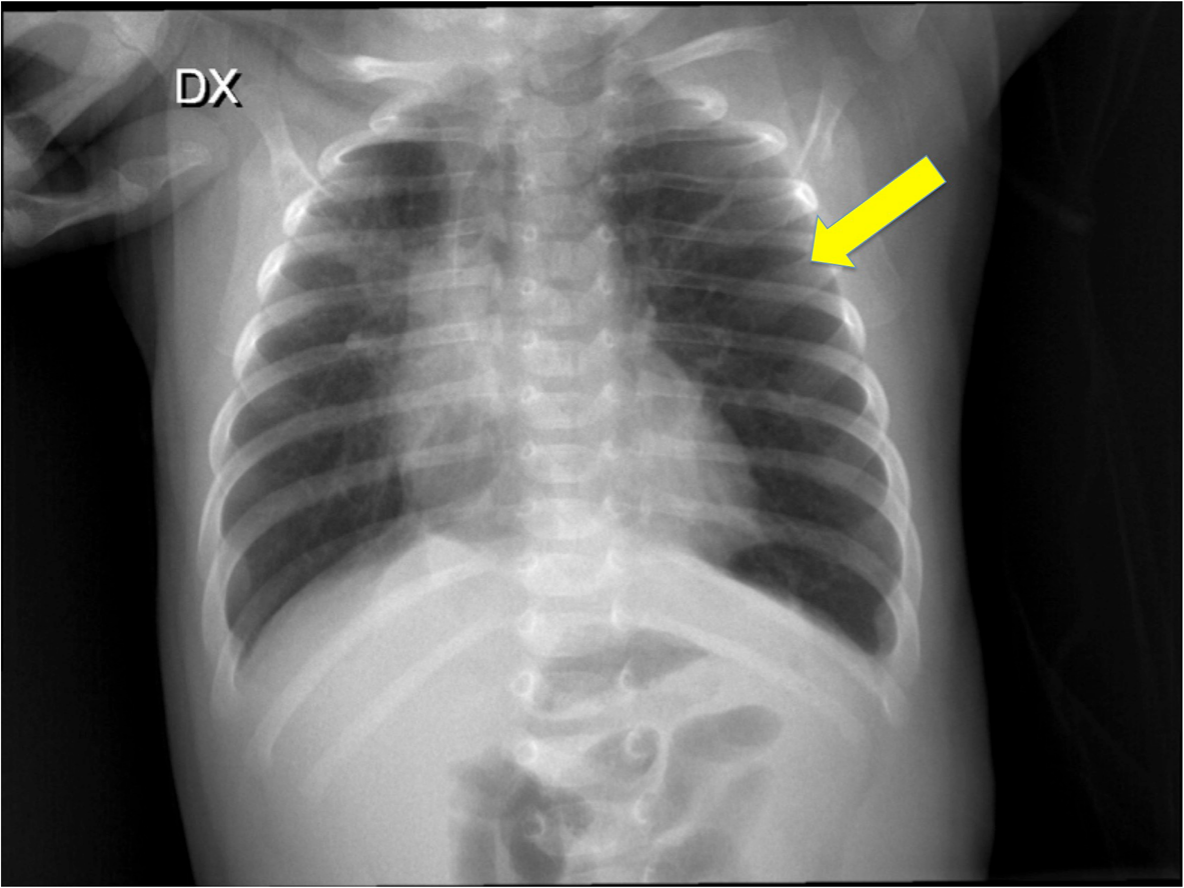
Chest X-ray at admission shows left pulmonary areas of hyperinflation (see arrows)

**Fig. 2:**
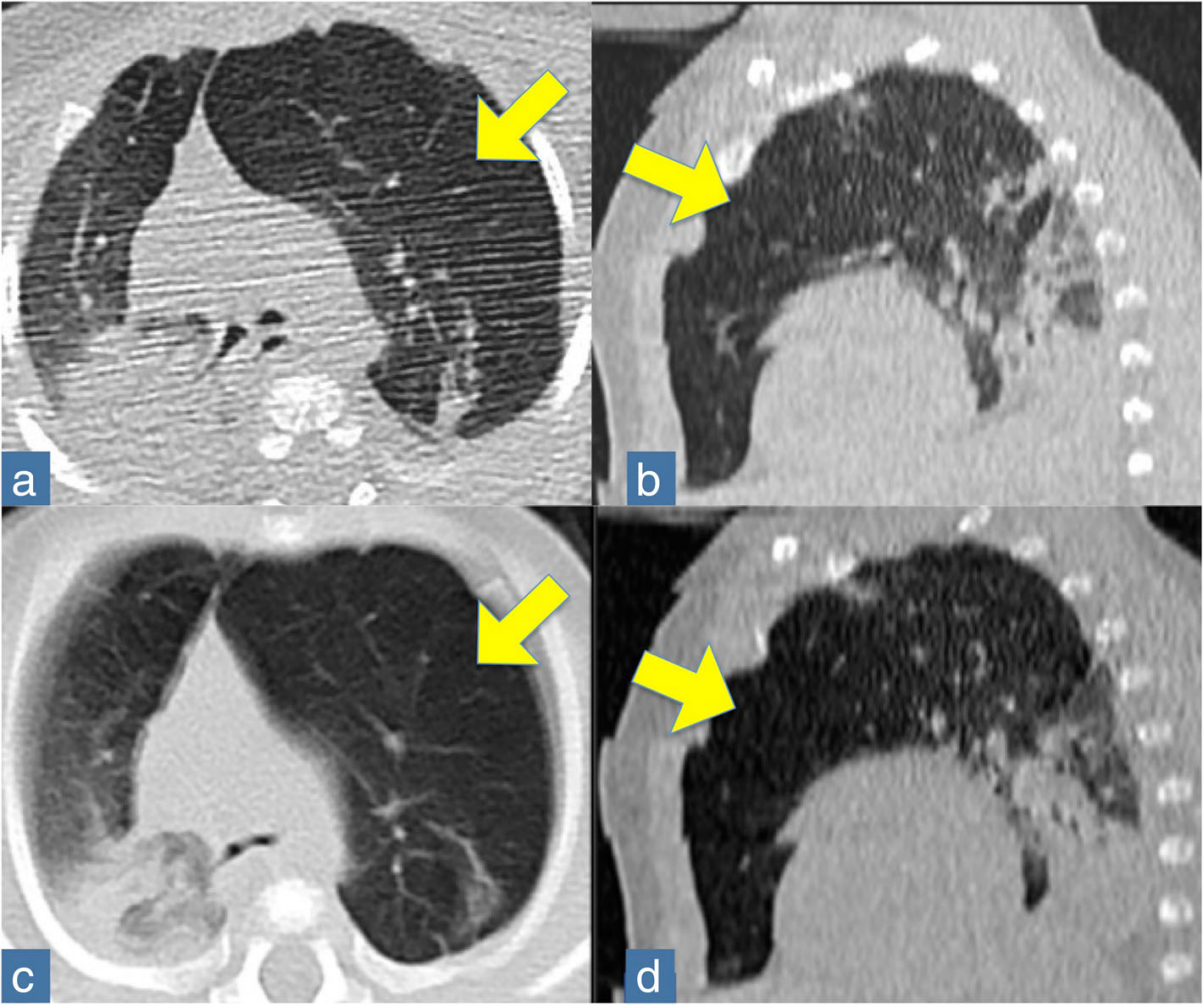
CT thorax at admission (Panels **a**, **b**) and two months later (Panels **c**, **d**). The arrows indicate the hyperinflation area. Panels a, c: axial position; Panel b, d: sagittal position

A chest X-ray (Fig. 1) and computed tomography (CT) scan (Fig. 2, Panels a, b) showed severe hyperinflation of the apical segment of the left lung and mediastinal shift to the right. A presumptive diagnosis of congenital lobar emphysema (CLE), including the lower lobe was made. After the stabilization of the subject’s respiratory conditions (non invasive respiratory support, fluid and electrolyte management, broad spectrum antibiotics, bronchodilatator), considering the inclusion of the superior lobe and the upper part of the lower lobe we decided to proceed with observation.

Two months later, the child’s condition deteriorated with worsening in respiratory distress; the child was unable to maintain saturation even with oxygen support. CT-angiography (Fig. 2, Panels c, d) was ordered and revealed a severe lobar emphysema of the anterior to the apicoposterior segment of the left upper lobe, with displacement of mediastinal structures to the right and compression of the right structures. A subsegmental atelectasis and areas of air trapping in the apicoposterior segment of the left lower lobe were also noted. Angiography showed peripheral pulmonary vascular attenuation and central pulmonary artery enlargement.

Surgery included a left upper lobectomy and segmental resection of the left lower lobe. The histopathology report was consistent with a generalized lung growth abnormality with alveolar enlargement and simplification.

Following surgery, multiple attempts to extubate the infant failed and he had a persistent oxygen requirement. Chronic respiratory failure ensued with progressive worsening of the ventilatory performance, necessitating continuous mechanical ventilation, with gradual support parameter adjustments and tracheostomy at age 12 months.

**Fig. 3:**
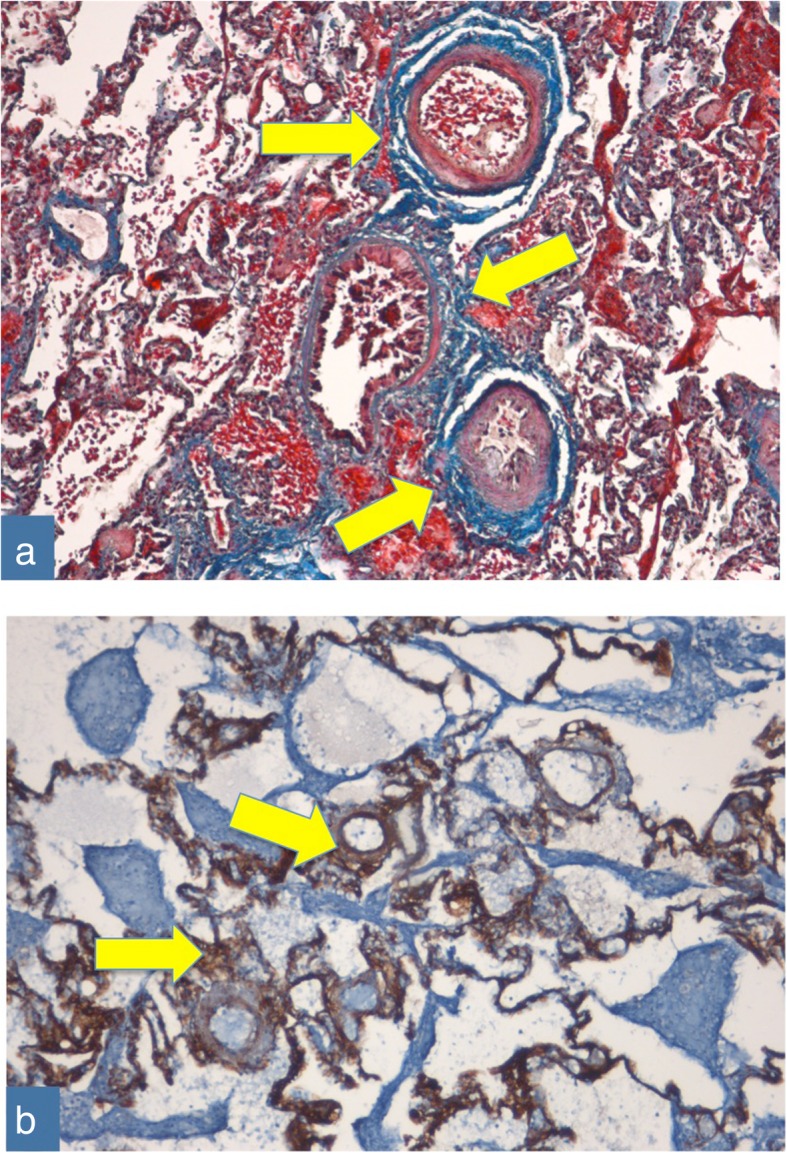
Histological features. In Panel **a,** areas in blue and the arrows indicate the perivascular and interstitial fibrosis and intra-alveolar hemorrhages (Azan-Mallory coloration, magnification 10x). In Panel **b**, areas in brown (Tenascin, magnification 10x) indicate where Tenascin was overexpressed, highlighting the extensive parenchymal fibrosis. TNC localization in the normal lung was un-detectable; TNC is specifically and transiently expressed upon tissue injury and down-regulated when tissue repair or scarring is concluded [38]

After prolonged multidisciplinary discussion, the decision to perform a surgical thoracoscopic lung biopsy was made in order to obtain additional data on the pathological pulmonary features for prognostic predictions and therapeutic decisions. Histopathology revealed alveolar enlargement, perivascular and interstitial fibrosis and intra-alveolar hemorrhages (Fig. 3).

Genetic testing was performed during the course of clinical care, after obtaining informed consent. Next generation sequencing on genomic DNA was performed using the NimbleGen SeqCap Target Enrichment kit (Roche) designed to capture several genes involved in pulmonary surfactant protein deficiency and skeletal abnormalities. A library was prepared following the manufacturer’s instructions and subsequently sequenced on an Illumina NextSeq550 instrument. Sequence data were carefully analyzed and the presence of all suspected variants were checked in the public databases (dbSNP, 1000 Genomes, and Exome Aggregation Consortium). The identified variants were confirmed by Sanger sequencing, following a standard protocol (BigDye® Terminator v3.1 Cycle Sequencing Kit,Life Technologies). No potentially causative variants were found in genes associated with cystic fibrosis, pulmonary surfactant protein deficiency or mutations in the *SETBP1* gene associated with Schinzel–Giedion syndrome (a rare autosomal dominant disorder that results in facial dysmorphism and organ and bone abnormalities).

Sequencing analysis showed a new mosaic frameshift variant, NM_001456.3: c.7391_7403del, p.Val2464Alafs*5 in the *FLNA* gene that was not present in the maternal blood DNA. This variant has not been previously reported in individuals with FLNA-related disorders, but can be classified as likely pathogenic (Class 4) according to the ACMG guideline and it is expected to cause disease. It is not present in any public databases, dbSNP (http://www.ncbi.nlm.nih.gov/projects/SNP/, 1000 Genomes Project (http://www.internationalgenome.org/), EVS (http://evs.gs.washington.edu/EVS/), ExAC (http://exac.broadinstitute.org/) and can be considered as a private variant.

The same mutation was identified in DNA from salivary and pulmonary mesenchymal stem cells of the patient [24].

**Fig. 4:**
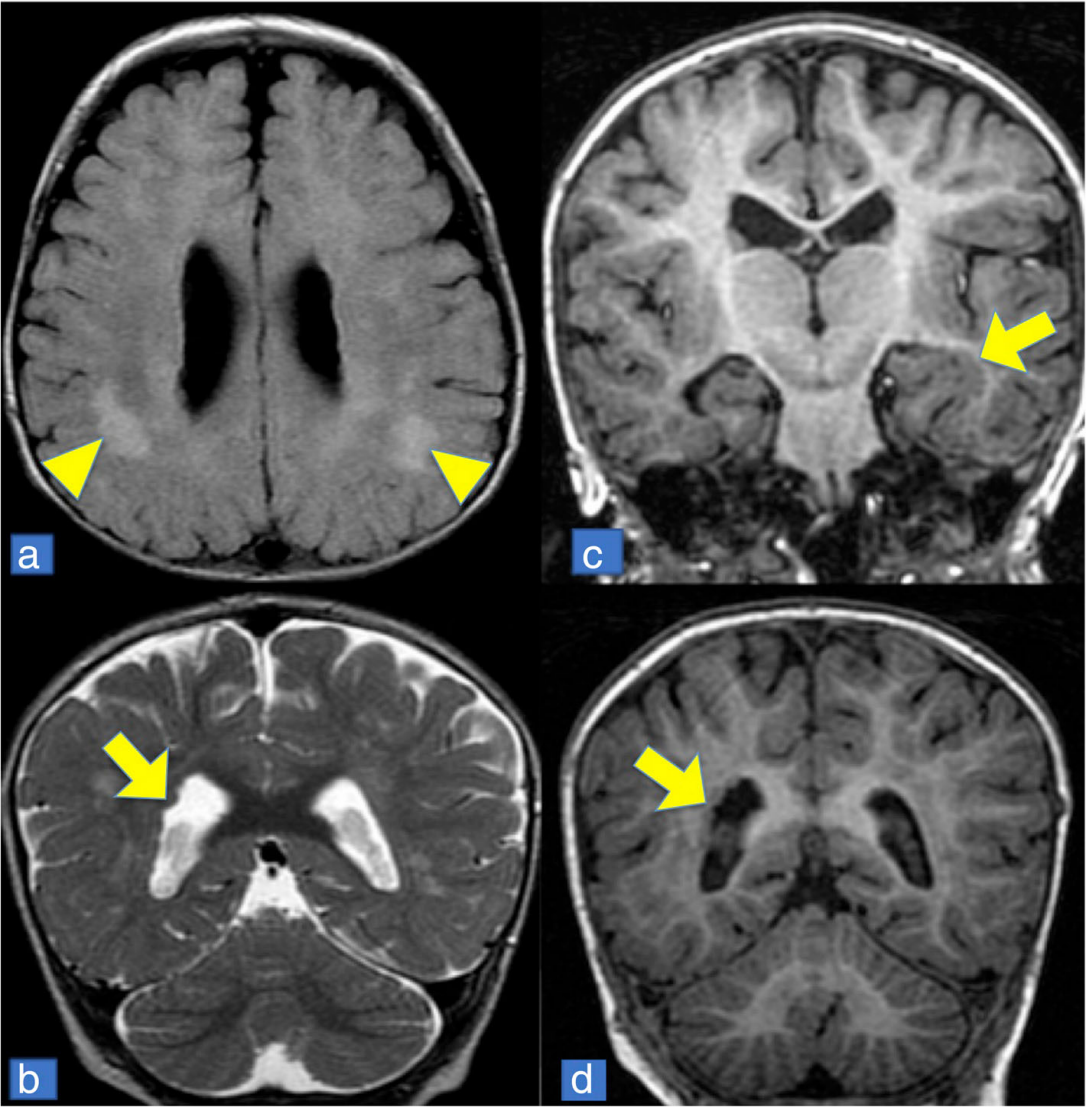
Brain MRI. Appearance of nodules (indicated by arrows) in periventricular grey matter heterotopia (images **b**, **e**, **d**), surrounding the left temporal horn and merging with the hippocampal cortex (image **c**). Supratentorial signal alterations with T2 and FLAIR hyperintense (images **a**, indicated by triangles) as in demyelinating lesions

Brain magnetic resonance imaging (MRI) depicted PVNH (Fig. 4), although the patient was not suffering from any neurological symptoms at this stage.

At 14 months follow-up, the patient requires mechanical ventilation and artificial nutrition to maintain his growth. Epilepsy and other neurological manifestations were not recorded.

## Discussion and conclusions

Filamin A is an actin-linking protein that regulates cell shape and migration of many cell types, including neuronal, vascular and cutaneous cells [15]. Filamin A is composed of three main functional domains: (1) a tandem N-terminal calponin-homology domain (CHD1 and CHD2), which confers F-actin binding properties; (2) 15 + 8 internally homologous Ig-like repeats separated by a short run with an unique sequence (hinge 1), important for flexibility; and (3) a second short run (hinge 2) followed by the C-terminal repeat 24, which are important for binding to a wide range of proteins and for dimerization [25]. Null mutations in the *FLNA* gene, lead to defects in neuronal migration, vascular function and connective tissue integrity. In contrast, gain-of-function missense mutations in this same gene produce a spectrum of malformations in multiple organ systems, especially the skeleton [26].

Here, we report the case of a male child in whom a new mosaic loss-of-function variant of the *FLNA* gene c.7391_7403del; p.Val2464Alafs*5 was found by next generation sequencing, resulting in significant lung disease characterized by emphysematous lesions and perivascular and interstitial fibrosis. The mutant allele frequency of this variant is estimated to be around 36% considering the numbers of sequence reads of the mutant and the wildtype alleles. This 13 bp deletion is predicted to result in a truncated protein that lacks the hinge 2 domain and repeat 24 probably leading to a loss of binding and dimerization ability that is essential for the FLNA function.

This report confirms an association between a *FLNA* gene mutation and lung disease. PNVH was observed and limb deformities were also present. There are 25 previous case reports in the literature on FLNA-related disorders with the pulmonary phenotype (Table 1) [2, 9, 13–22]. Lung diseases are associated with documented PNVH in 84% of the reviewed cases. The presence of cardiac co-morbidities, such as patent ductus arteriosis, valvular disease and aortic root dilatation, have also been reported [2–10, 13–15, 18]. Mutations in the filamin A gene are inherited in an X-linked (Xq28) dominant manner, with perinatal lethality in most males, whereas in female patients the prognosis depends on the severity of the associated cardiovascular abnormalities [20]. Of the previously published cases 21/25 (80%) were female (Table 1). Perinatal lethality occured in six of these reported cases (24%; 5 females and 1 male); in all cases, cardiopathies were also found [2–10, 13–15, 18]. As reported in Table 1, a large spectrum of *FLNA* mutations are detected in patients with pulmonary disease, including missense mutations [9, 13, 14, 19], nonsense mutations [2, 20], deletions [13, 15, 16, 21], duplications [13, 14, 18, 21], truncating mutations [17, 21], and frameshift mutations [14].

**Table 1:** Previous reports on *FLNA* gene variants associated with severe lung disorders

	Sex	Mutation	Principal clinical features	CT scan	Gestational age (weeks)	Age at presentation	Surgery	Documented PNVH	Outcome
Gerard-Blanluet et al., 2006 [19]	Male twins*	Missense mutation c.7922C > T (p.Pro2641Leu)	Severe bronchopulmonary dysplasia (BPD)	Not provided	26	26 weeks	None	Yes	Death of 1 infant at 8 mo; Follow-up until age 6 years (intercurrent respiratory infection)
*same family	Female*	Missense mutation c.7922C > T (p.Pro2641Leu)	Severe BPD	Not provided	24	24 weeks	None	Yes	Follow-up until age 2.5 years
De Wit et al., 2011 [9]	Female	Missense mutation c.220G > A (p.Gly74Arg)	Lobar emphysema (right middle lobe); bronchomalacia of right bronchial tree; frequent respiratory infections.	Severe lobar emphysema of right middle lobe; displacement of mediastinal structures	term	3 months	Lobectomy (right middle lobe)	Yes	Weaned from oxygen at 1 year, 7 months
Masurel-Paulet et al. 2011 [2]	Male	Mosaic nonsense mutation c.994delG (p.Lys331*)	Progression to severe lung disease	Congenital lobar emphysema;	term	3 months	Subtotal left upper lobectomy	Yes	Age 6 years. Supplemental oxygen during sleep
Clapham et al., 2012 [21]	Female	3′ *FLNA* deletion sparing first exon	Pulmonary emphysema involving multiple lobes	Not provided	39	2 months	None	Yes	Death at 7 months
	Female	3′ *FLNA* deletion and 5’*FLNA* duplication	Apical bullae of lung	Not provided	Not provided	ND	ND	Yes	Not provided
	Female	Deletion entire *FLNA* gene	Lobar emphysema	Not provided	Not provided	ND	ND	Yes	Not provided
Reinstein et al., 2013 [22]	Female (case F3)	Truncating mutation c.2193C > A (p.Tyr731*)	Pulmonary hypertension, focal hyperinflation with minimal patchy atelectasis	Not provided	Not provided	6 years	ND	Yes	Not provided
Lord et al., 2014 [17]	Female	Truncating mutation c.5683G3 > T (p.Gly1895*)	Progression to severe lung disease	Cystic pulmonary lesions;	36	24 days	none	Yes	Weaned from oxygen at 22 months
Lange et al., 2015 [20]	Male (case 29)	Mosaic non sense mutation c.7055-7070delCTTTTGCAGTCAGCCT (p.Ser2352*)	Severe progressive obstructive lung disease	Not provided	ND	38 years	Consideration of lung transplantation	Yes	Not provided
Eltahir et al., 2016 [18]	Female	Duplication c.3153dupC (p.Val1052Argfs*17)	Progressive lung disease	Lower lobe airspace disease, hyperinflation (right middle and left upper lobes)	36	2 months	PDA ligation	Not performed	Death at 15 months
Shelmerdine et al., 2017 [13]	Female	Deletion c.88delG (p.Ala30Profs*28)	Progressive lung disease	Left upper lobe and lower inflation; coarse septal thickening	36 + 5	3 months	PDA ligation	Not performed	Died at 9 months
	Female	Duplication c.6496dupA (p.lle2166Asnfs*3)	Multiple episodes of intercurrent pulmonary infections	Right upper and middle lobe over-inflation; coarse septal thickening; lower lobe atelectasis	term	7 months	Righ upper lobectomy, PDA ligation	Yes	Age 4 years
	Female	Missense mutation c.1528G > A (p.Ala510Thr)	Meconium aspiration	Right upper and left upper lobe over-inflation; coarse septal thickening; Lower lobe atelectasis	40 + 4	At birth	None	Not performed	Age 3 years. Therapy with bronchodilatator
	Female	Deletion c.2190_2193delTTAC (p.Tyr731Alafs*10)	Viral infections	Right upper and middle, left upper lobe over-inflation; Coarse septal thickening; Lower lobe atelectasis	38	3 months	None	Not performed	Age 6 years. Supplementary oxygen support
Burrage et al., 2017 [14]	Female	Duplication c.4596dupG (p.Ser1533Glufs*12)	Progressive lung disease	In all patients, severe pulmonary hyperinflation and hyperlucency with peripheral pulmonary vascular attenuation with parahilar and dependent lower lobe atelectasis and central pulmonary artery enlargement. In all patient, pulmonary arterial hypertension was also diagnosed.	39	2–4 months	PDA ligation	Yes	Lung transplantation in all. Five survivors at 19 months, 3 years, 4 years, 5.1 years, and 11.3 years respectively, post- follow-up. One died at 3 years
	Female	Missense mutationc.5290G > A (p.Ala1764Thr)	Progressive lung disease		40	neonatal	PDA ligation	Yes	
	Female	Duplication c.4446_4447dupAT (p.Leu1483Tyrfs*19)	Progressive lung disease		38	neonatal	PDA ligation	Yes	
	Female	Duplication c.4617_4618delGC (p.Leu1540Alafs*4)	Progressive lung disease		34	neonatal	PDA ligation	Yes	
	Female	Duplication c.6585dupT (p.Pro2196Serfs*3)	Progressive lung disease		39	neonatal	PDA ligation	Yes	
	Female	Missense mutation c.2807A > G (p.Lys936Arg)	Progressive lung disease		38	neonatal	PDA ligation	Yes	
Kinane et al. 2017 [15]	Female	Deletion c.6577delC (p.Arg2193Alafs*14)	Diffuse pulmonary abnormalities	Ground glass opacities, area of hyperacration, pulmonary hypertension	39	30 day	PDA ligation	Yes	
Sasaki et al. 2018 [16]	Female	Deletion c.1709_1712del (p.Val570Alafs*105)	Progressive lung disease	Diffuse bilateral groung-glass opacification throughout the lung, interstitial thickening, cystic changes	37	1 month	None	Yes	Died at 4 months
	Male	Splice site deletion c.6670-1delG	Several episodes of profound desaturation	Bilateral dependent and subsegmental atelectasis, scattered opacity, interstitial thickening	32	day of live 1	None	Yes	11 months, home oxigen
Our case	Male	Mosaic frameshift mutation c.7391_7403del; (p.Val2464Alafs*5)	Progressive lung disease	Lobar emphysema of the left upper lobe and a subsegmental atelectasis and areas of air trapping into the lower lobe	37	32 days	Left lobectomy Tracheostomy	Yes	15 months Mechanical ventilation

*FLNA* Filamin A; PNVH periventricular nodular heterotopia; CT computed tomography; PDA patent ductus arteriosus; ND Not provided

In these patients, the presentation of respiratory failure occurred at a median age of 1 month (range, birth to 72 months). However, one reported patient developed progressive obstructive lung disease at the age of 38 years [20]. The clinical presentation of lung involvement was variable, ranging from multiple episodes of intercurrent pulmonary infections [13], to progressive severe pulmonary disease [13, 14, 16–18, 20]. A variable outcome and management course were reported in the previously reported cases. In a limited number of patients, supportive therapy was successful [13, 16, 17, 19]. Surgical intervention in the form of lobar resection [2, 9, 13], as in our case, or lung transplantation, may be indicated in severe cases where supportative therapies are not successful [14, 20].

The pulmonary growth abnormality associated with FLNA deficiency consists of multilobar overinflation predominantly affecting the upper and lower lobes, with coarse septal thickening and varying lower lobe atelectasis with pruning of the peripheral pulmonary vasculature [27]. The role of FLNA in the development of lung disease is still not well elucidated. Considering that during respiration the lungs are subjected to mechanical forces and because FLNA plays important role in cell mechanosensing and mechanotransduction, abnormal FLNA interactions could affect pulmonary viscoelastic properties and disturb alveolar formation and growth [14, 28]. However, a role in T cell activation, interleukin production [29], inflammatory signaling [30] and interaction with the cystic fibrosis transmembrane conductance regulator [31] has also been proposed. Furthermore, the crucial role of FLNA action in mesenchymal migration, should not be excluded. Alterations in mesenchymal properties could be directly related to defects in cell migration during embryonic development and in pulmonary damage described in FLNA-defective patients [32]. Further studies are needed to investigate the functional role of tissue-resident lung mesenchymal stem cells in health and disease. Considering the successful use of stem cell therapy in the treatment of chronic progressive pulmonary disease in adults [31–37], future perspective stem cell treatment also in FLNA mutation-related lung disorders in children should be investigated. In conclusion, we report a novel mosaic loss-of-function variant of the *FLNA* gene associated with a severe lung disorder and PNVH. The lung disorder led to respiratory failure during infancy and these pulmonary complications may be the first sign of this disorder. Early recognition with thoracic imaging is important to guide genetic testing, neuroimaging and to define optimal timing of potential therapies, such as lung transplant in progressive lung disease [14].

## Abbreviations

CLE: Congenital lobar emphysema
CT: Computed tomography
FLNA: Filamin A
PVNH: Periventricular nodular heterotopia
